# Low surface recombination velocity in solution-grown CH_3_NH_3_PbBr_3_ perovskite single crystal

**DOI:** 10.1038/ncomms8961

**Published:** 2015-08-06

**Authors:** Ye Yang, Yong Yan, Mengjin Yang, Sukgeun Choi, Kai Zhu, Joseph M. Luther, Matthew C. Beard

**Affiliations:** 1 National Renewable Energy Laboratory (NREL), Golden, Colorado 80401, USA

## Abstract

Organic-inorganic hybrid perovskites are attracting intense research effort due to their impressive performance in solar cells. While the carrier transport parameters such as mobility and bulk carrier lifetime shows sufficient characteristics, the surface recombination, which can have major impact on the solar cell performance, has not been studied. Here we measure surface recombination dynamics in CH_3_NH_3_PbBr_3_ perovskite single crystals using broadband transient reflectance spectroscopy. The surface recombination velocity is found to be 3.4±0.1 × 10^3^ cm s^−1^, ∼2–3 orders of magnitude lower than that in many important unpassivated semiconductors employed in solar cells. Our result suggests that the planar grain size for the perovskite thin films should be larger than ∼30 μm to avoid the influence of surface recombination on the effective carrier lifetime.

## Abstract (web summary)

Surface recombination velocity can have a major impact on solar cell performance. Here, Yang *et al*. measure surface recombination dynamics in perovskite single crystals using broadband transient reflectance spectroscopy. Grain size is crucial to avoid the effects of surface recombination on carrier lifetime.

## Graphical abstract

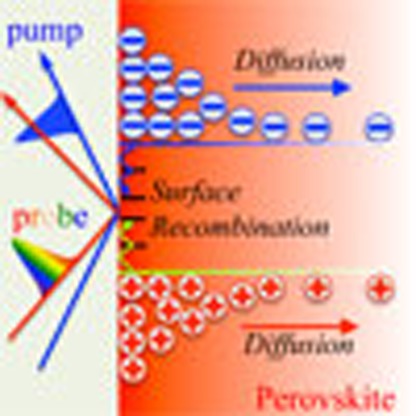

Carrier recombination at semiconductor surfaces has a detrimental impact on solar cell performance because it decreases carrier lifetimes reducing the attainable short-circuit current and open-circuit voltage^1^. Therefore, surface passivation is a necessary component in achieving high efficiency solar energy conversion^2^. The knowledge of surface recombination can also provide guidance for optimizing the grain size in polycrystalline solar cells^3^. Solution processed lead halide based solar cells are undergoing impressive improvements in power conversion efficiency^4^,^5^,^6^,^7^,^8^. While thorough characterization in transport carrier parameters such as mobility and bulk lifetime shows sufficient characteristics^9^,^10^,^11^,^12^,^13^,^14^,^15^,^16^, surface recombination has not been studied.

Here we directly probed the carrier dynamics at the surface of CH_3_NH_3_PbBr_3_ perovskite single crystals using broadband transient reflectance (TR) spectroscopy. The lead bromide perovskite single crystals are chosen because their sizes are sufficiently large for optical measurements and their native surfaces do not require further polishing or treatments. In the TR measurement, optical excitation of the perovskite single crystal can modulate the reflectance, *R*, near the bandgap and the relative reflectance change (Δ*R*(*ℏω*)/*R*) is recorded by a white-light continuum. We quantitatively reproduce the spectrum using Kramers–Kronig integration of excitonic absorption bleach and find a linear relationship between Δ*R*(*ℏω*)/*R* and the total photoexcited carrier density, *N*, which includes both free carriers and excitons. Pumping at different photon energies with various intensities, we find that kinetics of Δ*R*(*ℏω*)/*R* is only sensitive to *N* near the surface, and is modulated by surface recombination and carrier diffusion that transports carriers away from the surface. The surface recombination velocity (SRV) and carrier diffusion coefficient (*D*) are found to be 3.4±0.1 × 10^3^ cm s^−1^ and 0.27±0.01 cm^2^ s^−1^, respectively, by global fitting of a diffusion model to Δ*R*(*ℏω*)/*R* kinetics collected at different excitation energies.

## Results

### Single crystal characterization

**Figure 1:**
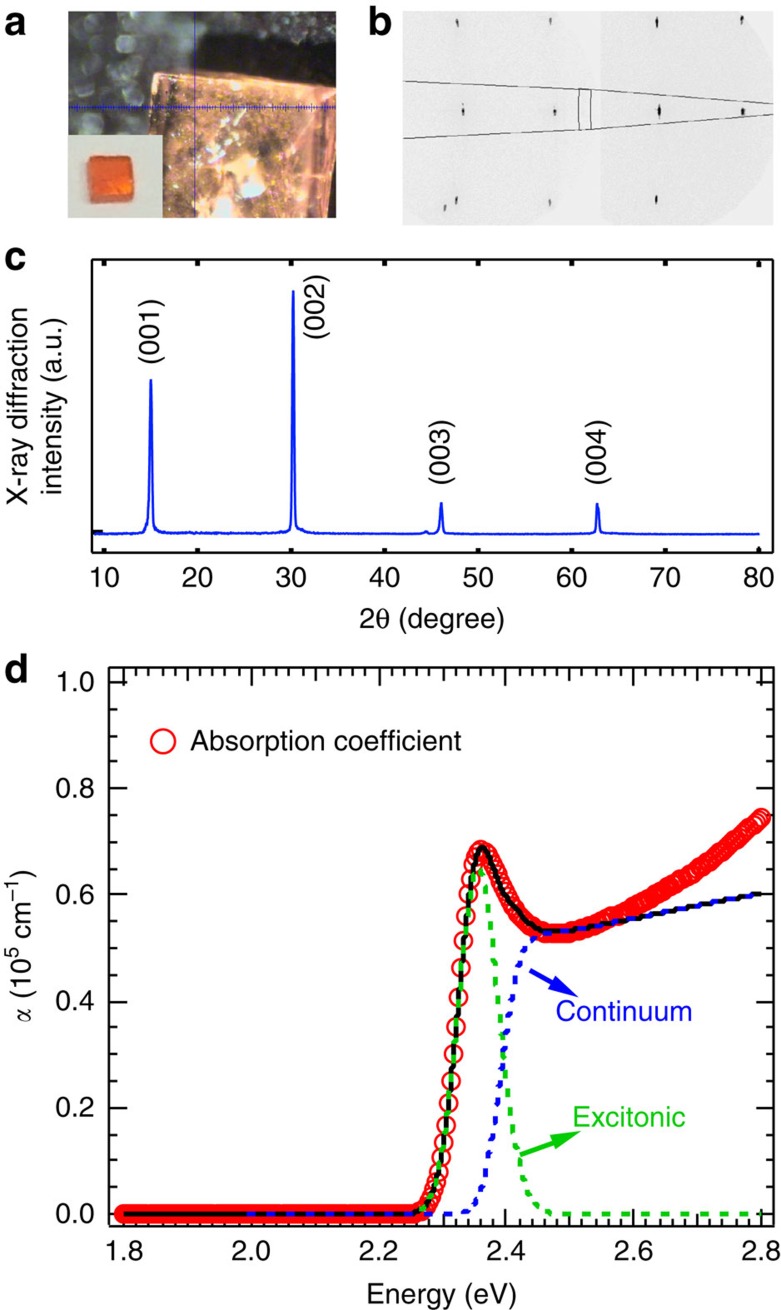
Structure characterization and optical absorption spectrum. (**a**) The microscopic image of the single crystal. The cross-hair indicates that the X-ray beam is located at the front face. Inset is the photograph of the single crystal. (**b**) X-ray diffraction pattern measured by two-dimensional area detector. The right and left frame are corresponding to 2*θ* centred at 30 and 60°, respectively. (**c**) Integration of the X-ray diffraction pattern. The diffraction intensity is integrated in the region of *χ*=90±5°, indicated by the black lines in panel **b**. *χ* is the angle between the plane determined by the incident and diffracted beams and the plane of the front face. (**d**) The absorption coefficient (red circles) of the CH_3_NH_3_PbBr_3_ perovskite single crystal obtained from ellipsometry measurement. The black-line is the modelled absorption coefficient with excitonic (green-dash line) and continuum (blue-dash line) components.

The solution-grown CH_3_NH_3_PbBr_3_ single crystals studied here have dimensions of ∼1.4 × 1.4 × 0.7 mm^3^ (inset of Fig. 1a). We measured the X-ray diffraction using a Bruker D8 Discover diffractometer with two-dimensional area detector. The X-ray beam is located at the front face of the crystal (Fig. 1a). A diffraction pattern of isolated spots, rather than continuous arcs (Debye ring), is observed (Fig. 1b) and it is caused by X-ray diffraction from oriented lattice planes, consistent with high quality single crystals. To obtain the Miller index of the front face, the diffraction intensity is integrated for the region where the crystal front face and the plane determined by the incident and diffracted X-ray beams are perpendicular (inside the black lines in Fig. 1b, where *χ*=90±5°). Such an integration produces intensity peaks that correspond to diffraction from the lattice planes that are parallel to the front surface of the single crystal (Fig. 1c). These diffraction peaks can be assigned to the family of (001) planes of a cubic phase perovskite crystal, suggesting that the front face belongs to this family of planes. The optical measurements in this work were all conducted with incident light on the front surface. We carried out similar X-ray diffraction measurements on the side face of the crystal and find that it also belongs to the family of (001) planes (Supplementary Fig. 1). The integration of the whole X-ray diffraction pattern (*χ* integrated from 0 to 180°) produces the diffraction peaks corresponding to other lattice planes (Supplementary Fig. 1).

### Simulation of optical absorption near bandedge

The absorption coefficient (Fig. 1d), determined from ellipsometry (Supplementary Fig. 2), shows a prominent absorption band at 2.35 eV attributed to excitonic absorption^17^,^18^. We simulate the spectrum near the bandedge according to Elliott's formula^19^,^20^:


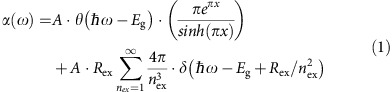


where *A* is a constant related to the transition matrix element, *ω* is the frequency of light, θ is the step function, *E*_g_ is the bandgap, *x* is defined as 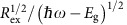 where *R*_ex_ is the exciton binding energy, *n*_*ex*_ is the principal quantum number and *δ* denotes a delta function. The first term describes the continuum state absorption and the second term is for excitonic states. We modelled the absorption (black line, Fig. 1d) accounting for inhomogeneous broadening by convolving with a Gaussian function (see Supplementary Table 1 for fitting parameters). We neglect the excitonic transitions with *n*_*ex*_ larger than 6 because the oscillator strength decreases as 1/n_ex_^3^. We find the best-fit values of *R*_ex_ and *E*_g_ to be 41.6 meV and 2.394 eV with <0.1 meV fitting uncertainty. The exciton binding energy found here is about half of the calculated value^18^. For photon energies greater than ∼2.55 eV, the absorption differs from the model because higher energy bands are not included.

### Transient reflectance spectroscopy

**Figure 2:**
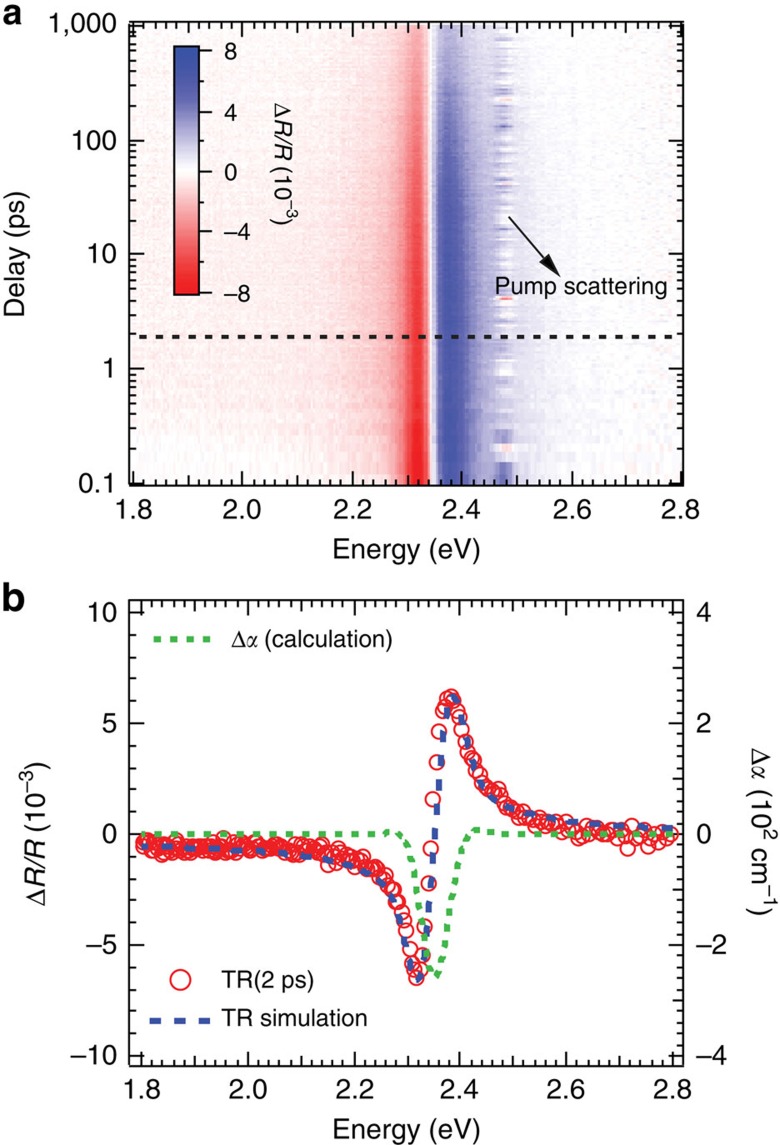
TR spectra and simulations. (**a**) Pseudocolor representation of the transient reflectance spectra. (**b**) The transient reflection spectrum at delay time of 2 ps (dotted line in panel **a**) and the corresponding simulation (blue-dash line). The calculated change in absorption coefficient is shown as the green-dash line.

For the pump-probe TR measurements, the pump is monowavelength with photon energy of 2.48 eV and the probe is broadband with the spectrum ranging from 2.95 to 1.50 eV. The pump penetration depth is calculated as 190 nm from the absorption coefficient while the effective detecting depth is ∼*λ*/*4πn* (*n* is refractive index)^21^ and is ∼18 nm for probe photon energies near bandgap. The pseudocolor image of the TR spectra is shown in Fig. 2a with a representative spectrum at 2 ps (Fig. 2b). There are two anti-symmetric peaks centred at 2.35 eV. At longer delays, the magnitude decreases while the spectral shape persists. Since Δ*R*(*ℏω*)/*R* is small (∼0.005) and the refractive index *n*(*ℏω*) is much larger than the extinction coefficient *k*(*ℏω*) in the spectral region of interest (Supplementary Fig. 2), Δ*R*(*ℏω*)/*R* can be approximated as:


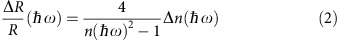


where Δ*n* is the frequency-dependent photon induced change in refractive index associated with a change of absorption coefficient, Δ*α*, and is calculated using the Kramers–Kronig transformation^22^:


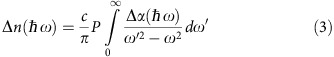


where *c* is the speed of light, and *P* means Cauchy principal value of the integral. The photo-induced Δ*α* includes both bleach of the exciton absorption and continuum band absorption. However, at low excitation levels, the bleach of continuum band absorption, caused by band filling, is not important because of the low occupation probability. Thus, Δ*α* is dominated by the bleach of exciton absorption resulting from photo-induced carriers (free carriers and excitons) due to the phase-space filling effect, and can be expressed as^23^,^24^:


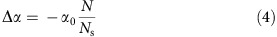


where *α*_0_ is the steady state exciton absorption coefficient (green-dash line, Fig. 1d), *N* is the total carrier density, equal to the excitation density, and *N*_s_ is a saturation density (defined to be the carrier density for which 50% of exciton absorption is bleached) and is related to the exciton Bohr radius (note, equation 4 is only valid when *N*<<*N*_S_ (ref. 25)). We are able to closely simulate Δ*R*(*ℏω*)/*R* (blue-dash line, Fig. 2b) by substituting equations (4) and (3) into equation (2) with fitting parameter, *N*_S_. The simulation reveals Δ*α* (green-dash line, Fig. 2b) and the best-fit value of *N*_S_ (6.0 × 10^18^ cm^−3^). These equations imply that Δ*α* and Δ*R*(*ℏω*)/*R* are proportional to the excitation density, *N*, for *N<<N*_S_.

**Figure 3:**
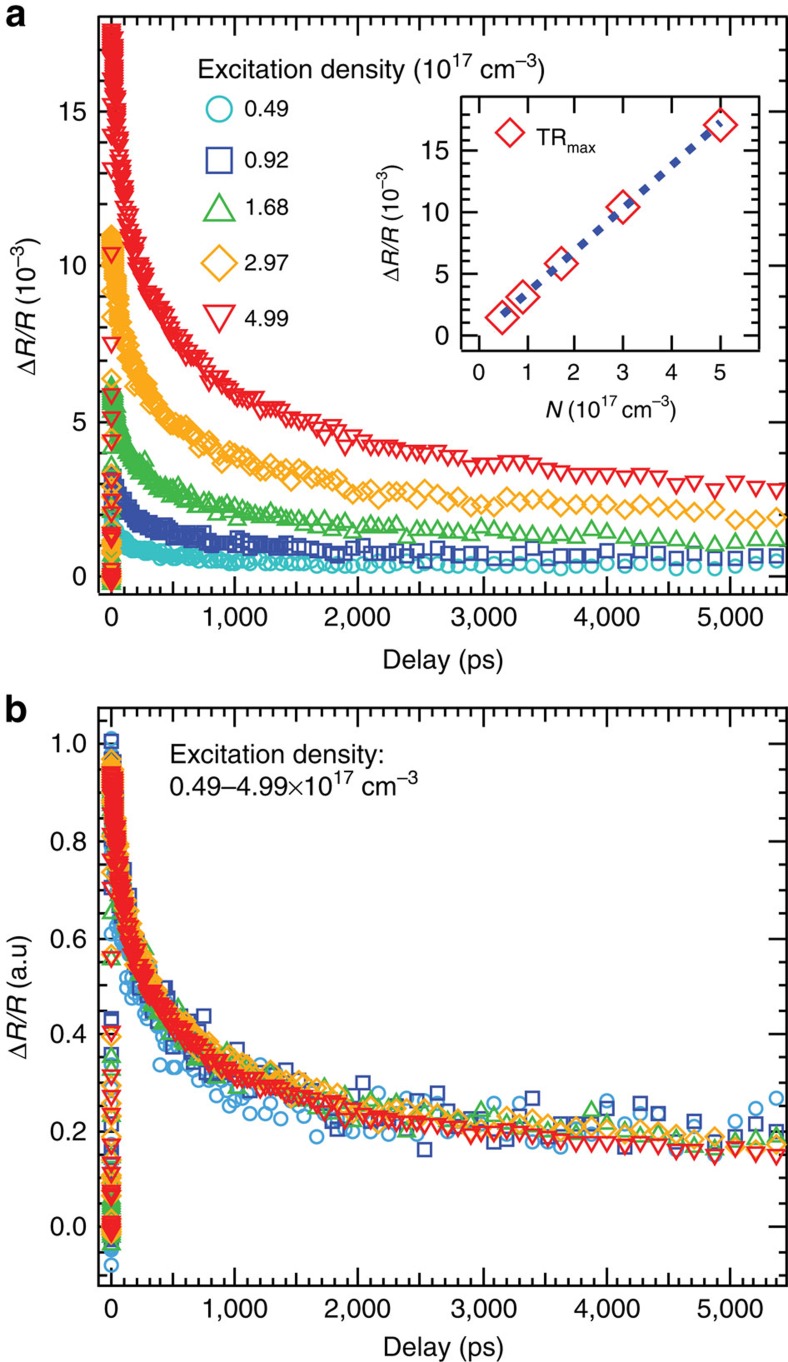
Excitation intensity dependent TR kinetics. (**a**) The kinetics of transient reflectance recorded at 2.38 eV for different excitation intensities. The pump energy is 2.48 eV. Inset is the plot of maximum amplitude as function of excitation intensity. (**b**) The normalized kinetics shown in panel **a**.

The TR measurements were conducted under various pump intensities and the kinetics (probe photon energy of 2.38 eV) are shown in Fig. 3a. The kinetic traces do not display single-exponential behaviour. The decay is faster at early delay and slower at later delay. The initial magnitude shows a linear relationship with excitation density (inset Fig. 3a). At higher excitation levels, the bleach of the continuum band absorption due to the band filling begins to contribute and the exciton contribution saturates, resulting in a Δ*α* (as well as TR response) that is no longer linear with the excitation intensity^14^. In this work, excitation intensities are all controlled to be in the linear region (*N*∼0.02*N*_S_) so the photo-induced exciton absorption bleach dominates the TR response.

Figure 3b contains the normalized data from Fig. 3a, showing that TR kinetics for a given excitation energy are independent of excitation density, over 1 order of magnitude. The decay of the TR kinetics suggests the carrier depopulation and/or diffusion out of the probing region. In the bulk, the depopulation from first-order radiative and/or Shockley–Read–Hall (nonradiative) recombination is independent of the excitation density. However, they are excluded because the first-order bulk recombination occurs on a much longer time scale (∼31 ns, measured by time-resolved photoluminescence, Supplementary Fig. 3). The decay of TR kinetics is therefore attributed to the surface recombination and the carrier diffusion from the surface into the bulk. Since no electric filed is applied, the carriers are expected to diffuse together as either uncorrelated electron-hole pairs or excitons to maintain the charge neutrality.

### Diffusion and surface recombination model

**Figure 4:**
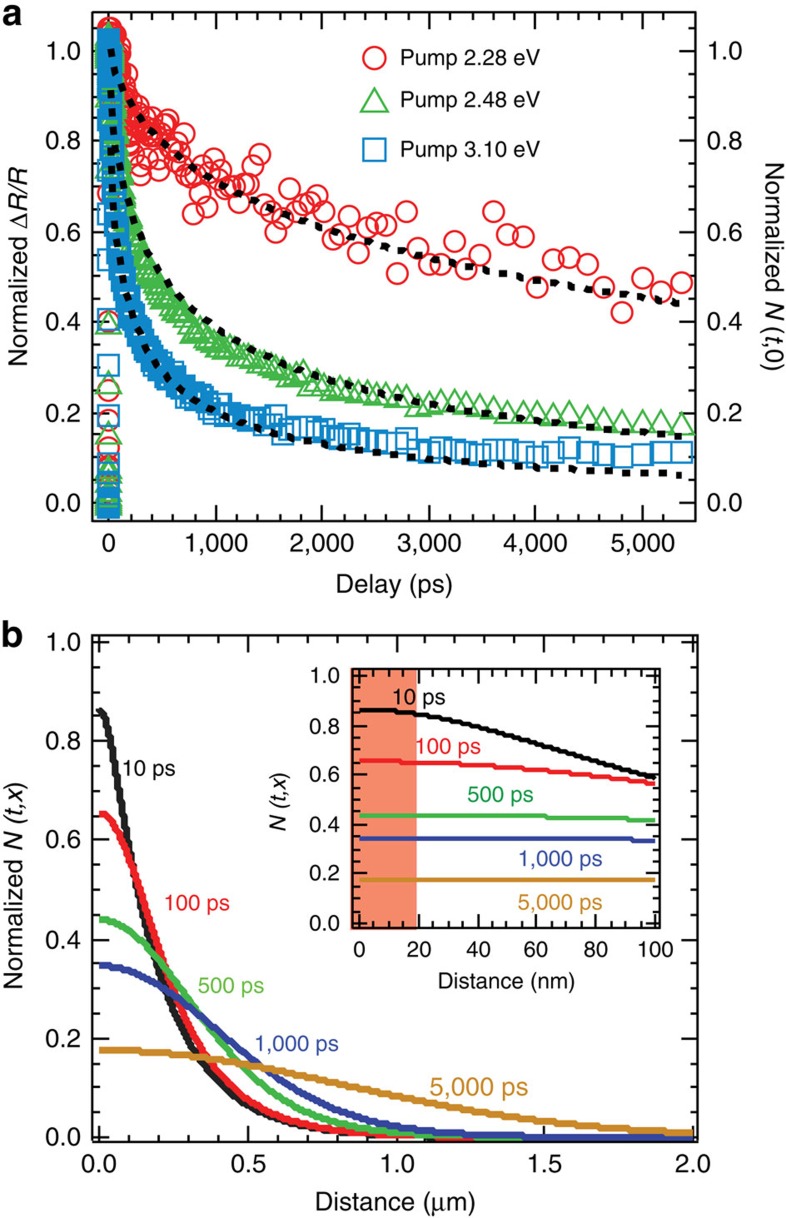
Excitation energy dependent TR kinetics and carrier density distribution profiles. (**a**) The normalized TR kinetics recorded at 2.38 eV for three different pump energies. The normalized surface carrier density dynamics shows the same decay trend as the TR kinetics. The black dashed lines represent the global fitting based on the carrier diffusion model. (**b**) Normalized carrier density distribution profiles for 2.48 eV pump at indicated delays in the single crystal. Inset shows the distributions within 100 nm from the surface, and the red shade represents the probe (at 2.38 eV) detection depth.

To explore Δ*R*/*R* recovery kinetics, we varied the excitation photon energy. In addition to 2.48 eV the sample was excited at 3.10 and 2.28 eV with corresponding penetration depths of 80 nm and 3.7 μm. We confirmed for each photon energy that TR kinetics are independent of excitation density (Supplementary Figs 4 and 5). However, the Δ*R*/*R* dynamics exhibits a clear dependence on the excitation energy (Fig. 4a). In Fig. 4a the data for different excitation energies are normalized for comparison and we find that the kinetics decays faster for larger excitation energies corresponding to shorter penetration depths. Because of the linear relationship between Δ*R*/*R* and excitation density discussed above, the TR kinetics follow the total carrier dynamics in the effective detecting region. To understand the relationship between carrier density and surface recombination, we use a one-dimensional diffusion model that includes surface recombination to reproduce the TR kinetics. The analytical expression for the normalized carrier density distribution as function of time is^26^,^27^:


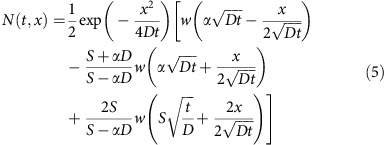


where *t* is delay time, *x* is distance from surface, *D* is the ambipolar diffusion coefficient, *α* is absorption coefficient at the excitation energy, *S* is surface recombination velocity, and *w*(z)=exp(*z*^2^)[1−erf(*z*)]. Since the pump penetration depths are always larger than effective detecting depth, the carrier density in detecting region can be approximated as the carrier density evaluated at the surface, *N*(*t*,0)^28^,^29^ (the validity of this approximation is explored below). A non-linear global fitting routine is used to model the three kinetic traces simultaneously by calculating *N*(*t*,0). In the fitting procedure, the absorption coefficient, *α*, at each pump-photon energy is determined from the ellipsometry (Supplementary Fig. 2), and *S* and *D* are free fitting parameters. The best-fit results are shown in Fig. 4a with best-fit parameters of *S*=3.4±0.1 × 10^3^ cm s^−1^ and *D*=0.27±0.01 cm^2^ s^−1^. To directly compare with reported value, we calculated the ambipolar mobility as 10.8 cm^2^ *V*^*−1*^ *s*^*−1*^ from *D* via the Einstein relation. Our value is consistent with that determined from Hall effect measurement of CH_3_NH_3_PbBr_3_ single crystals^9^, ∼1–10 times larger than that obtained for CH_3_NH_3_PbX_3_ (X=Br and I) polycrystalline films^11^,^12^,^15^,^16^,^17^, and ∼10 times smaller than that obtained from time-of-flight measurement of the perovskite single crystals^9^,^10^. The SRV determined here is ∼2–3 orders of magnitude smaller than those in other important semiconductors (before surface passivation) such as GaAs (*S*∼8.5 × 10^5^ cm s^−1^)^30^, GaP (*S*∼2 × 10^6^ cm s^−1^)^31^, InP (*S*∼1.5 × 10^5^ cm s^−1^)^26^, p-Si (*S*∼2.4 × 10^5^ cm s^−1^)^32^, n-Si (*S*∼1.2 × 10^6^ cm s^−1^)^32^, and CdTe (*S*∼5 × 10^5^ cm s^−1^)^33^.

In Fig. 4b, we display the calculated carrier distributions at various delay times according to equation (5) for 2.48 eV excitation. The carrier density decreases near the surface and increases in the bulk due to diffusion. The inset shows *N*(*t*,*x*) close to surface, where the red shaded region represents the effective detecting depth (∼18 nm). The carrier distribution is nearly uniform in this regime, confirming that the carrier density that affects Δ*R*/*R* can be safely approximated by the surface density.

## Discussion

We measured TR kinetics for several different single crystals obtained from the same growth method and for different faces of the same crystal (Supplementary Fig. 6) and find a high degree of reproducibility of our measurement. For single crystal semiconductors, midgap states created by surface defects are primarily responsible for surface recombination. Theoretical calculations suggest that defects at grain boundary or surfaces for CH_3_NH_3_PbX_3_ (X=Br and I) perovskite in either cubic or tetragonal phase cause very few midgap states^34^,^35^,^36^,^37^, which may explain the low SRV determined here. Compared with the bromide perovskite, the iodide perovskite likely has less midgap states due to the narrower bandgap, and thus we expect an even lower SRV (ongoing measurements).

**Figure 5:**
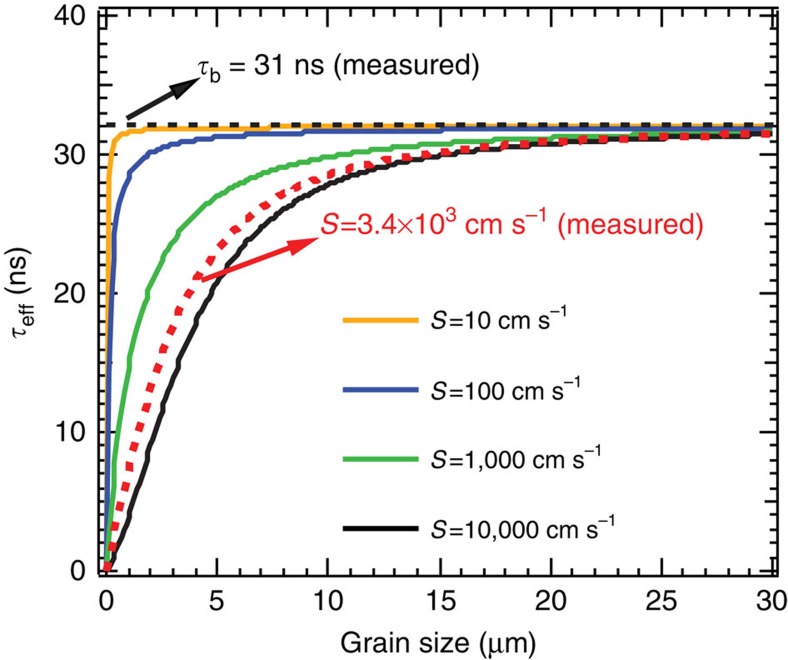
Effective lifetime (*τ*_eff_) in polycrystalline films. *τ*_eff_ are plotted as function of the grain size for various surface recombination velocity. The black dashed line represents the measured bulk lifetime. The red-dash line is effective lifetime as function of grain size calculated according to the SRV determined in the perovskite single crystals.

For polycrystalline thin films, the surface recombination at grain boundaries can determine the effective carrier lifetime. To minimize the impact from surface recombination, the grain size cannot be too small. The effective lifetime as function of grain size with a given surface recombination velocity (*S*) is approximated by^38^:


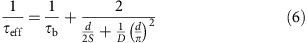


where *τ*_b_ is the bulk carrier lifetime measured above, *d* is planar grain size. The effective lifetimes, *τ*_eff_, are calculated with *D*=0.27 cm^2^ s^−1^ plotted in Fig. 5 for various SRV. We find that, ultra low SRV (*S*∼10–100 cm s^−1^) is required to make *τ*_eff_ approach *τ*_b_ when *d*<5 μm, suggesting that surface passivation will be necessary for the perovskite cells with grain size smaller than 5 μm. For unpassivated perovskites (*S*∼10^3^–10^4^ cm s^−1^), the surface recombination influence on the *τ*_eff_ can be eliminated when the grain size is larger than ∼30 μm. The effective lifetime curve calculated with the measured *S* and *D* (Fig. 5, red-dash curve) shows close correspondence with the reported trend of the perovksite solar cell efficiency versus apparent grain size, in which the efficiency was boosted by 10 times as the grain size increased from 1 to ∼20 μm while efficiency growth slope became much slower when the grain size was larger than ∼20 μm (ref. 4).

In solar cells, charge recombination across interfaces between active layers and charge transporting layers can also impact the performance. Recently, Tress *et al*. demonstrated that interfacial charge recombination could be suppressed by optimizing the thickness of the hole transporting layer in perovskite solar cells^39^. In this case, the open-circuit voltage is limited by the nonradiative bulk and surface recombination^40^. Considering the long bulk lifetime, surface recombination likely limits the open-circuit voltage for perovskite solar cells. Thus, lowering surface recombination should enhance the open-circuit voltage.

It should be noted that the surface recombination measured here is from a single crystal surface without post treatment. However, in working solar cells, the surfaces could be unintentionally passivated by contacting with charge transport layers^41^ or forming a lead halide over layer^42^, which may lead to a reduction of surface recombination. The passivation dependent surface recombination is beyond the scope of this paper, and will be the subject of the future work.

In summary, we conducted TR measurements for perovskite single crystals. The TR spectra are quantitatively described by excitonic absorption bleach, and carrier dynamics is attributed to surface recombination and carrier diffusion from the surface into the bulk. By simultaneously fitting the carrier decay for different excitation energies, SRV and diffusion coefficient are obtained. The measured SRV is ∼2–3 order of magnitude smaller than that in many semiconductors used for solar cells. Our result also suggested that the grain size in polycrystalline perovskite film should be larger than ∼30 μm or grain boundaries should be further passivated to eliminate the surface recombination influence on effective lifetime.

## Methods

### Transient reflectance spectroscopy

Femtosecond pump-probe TR experiments were performed based on a regeneratively amplified Ti:sapphire laser system that produces ∼4 mJ laser pulses at 800 nm with 1 KHz repetition rate. The pumps for TR are generated by an optical parametric amplifier pumped by 800 nm fundamental pulses (∼1.5 mJ per pulse), which is chopped at a rate of 500 Hz and attenuated by neutral density filter wheels. The broadband probe pulses (420–830 nm) are generated by focusing 800 nm light into a sapphire crystal. The probe pulses are delayed in time with respect to the pump pulses using a motorized translation stage mounted with a retroreflecting mirror. The pump and probe are spatially overlapped on the surface of the sample, and the probe pulses are directed to the multichannel complementary metal–oxide–semiconductor sensor. The size of the focused spot at the sample position for probe and pump beams are 180 and 560 μm, respectively. The total pump-photon flux is determined by measuring the pump power after a pinhole with radius of 200 μm at the sample position. The input photon flux is obtained by subtracting the reflected photon flux from the total photon flux. The excitation density is calculated as the ratio of input photon flux to the effective depth that is 1 over absorption coefficient at pumping energy.

### Sample preparation

To a 5 ml dimethylformaide solvent, PbBr_2_ (183 mg, 0.5 mmol) and CH_3_NH_3_Br (56 mg, 0.5 mmol) were dissolved. The mixture solution was heated slightly to obtain a transparent solution. This solution was further filtered through a compacted celite column. The filtrate was collected. Two millilitre of this solution was transferred into an inner vial (5 ml in total vial volume) that was placed in a larger outer vial (25 ml in total volume) with 5 ml of toluene inside. Finally the outer vial was carefully sealed. The diffusion of toluene from outer vial into the inner vial was slow and the crystallization process was maintained in dark and undisturbed environment for at least three days. The orange block-shaped single crystals were obtained and characterized by X-ray diffraction.

## Additional information

**How to cite this article:** Yang, Y. *et al*. Low surface recombination velocity in solution-grown CH_3_NH_3_PbBr_3_ perovskite single crystal. *Nat. Commun.* 6:7961 doi: 10.1038/ncomms8961 (2015).

## Supplementary Material

Supplementary Information Supplementary Figures 1-6 and Supplementary Table 1
