# Supplementary material for: Low surface recombination velocity in solution-grown CH3NH3PbBr3 perovskite single crystal
Source: Nat Commun. 2015 Aug 6;6:7961. doi: 10.1038/ncomms8961 (PMC4918347; doi:10.1038/ncomms8961)
Supplement: Supplementary Information — Supplementary Figures 1-6 and Supplementary Table 1 [file ncomms8961-s1.pdf]

Supplementary Figures.

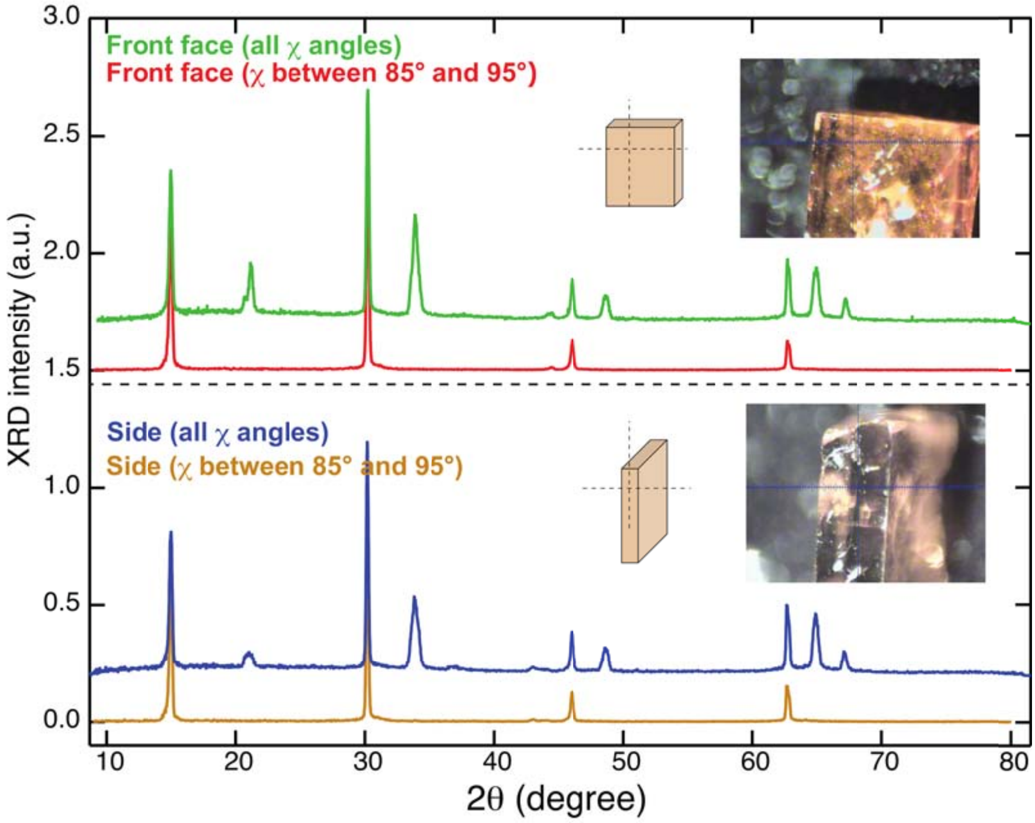

Supplementary Figure 1. Crystal Structure Characterization. Upper Panel: XRD patterns measured from the front face of the single crystal. The inset cartoon and photograph with crosshairs indicate the X-ray beam is diffracted by the front face of the crystal. The green and lower red XRD patterns are obtained by integrating the diffraction intensity over all χ values and 85° < χ <95°, respectively. χ is defined as the angle between crystal surface and the plane determined by the incident and diffracted X-ray beams. The green diffraction pattern only gives the information of the lattice planes that parallel to the front face, while the red XRD pattern includes more lattice planes. Lower Panel: XRD patterns measured from the side face of the single crystal. The inset cartoon and photograph with crosshairs indicate the X-ray beam is diffracted by the side face of the crystal. The blue and orange XRD patterns are obtained in the same way as the green and red pattern in the upper panel. The labeled vertical black lines are the calculated XRD pattern for the cubic phase $CH_3NH_3PbBr_3$ perovskite. The measured diffraction peaks match well with calculated ones except the peak intensity. The discrepancy in peak intensity is because in the single crystal the lattice planes are single oriented and in the powder all the lattice planes are in random orientation. The comparison between all measured XRD patterns and the calculated one suggests that the crystal front and side faces all belong to the family of (001) plane and the single crystal is in cubic phase.

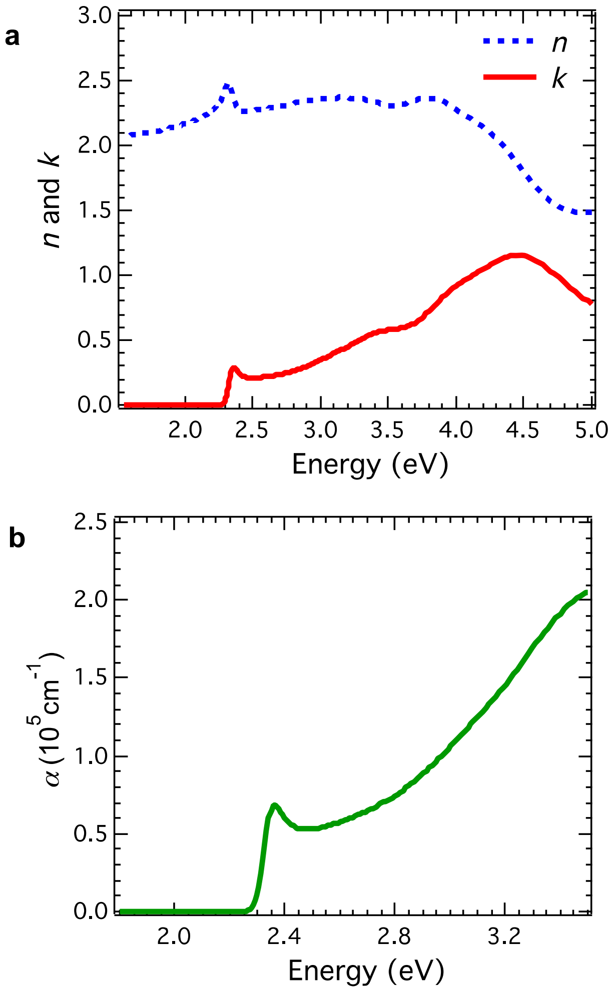

Supplementary Figure 2. Complex refractive index and absorption coefficient. (a) The complex refractive index measured by ellipsometry. The real and imaginary components are denoted as n (refractive index) and k (extinction coefficient). (b) The absorption coefficient, α, calculated from $\alpha=4\pi k/\lambda$.

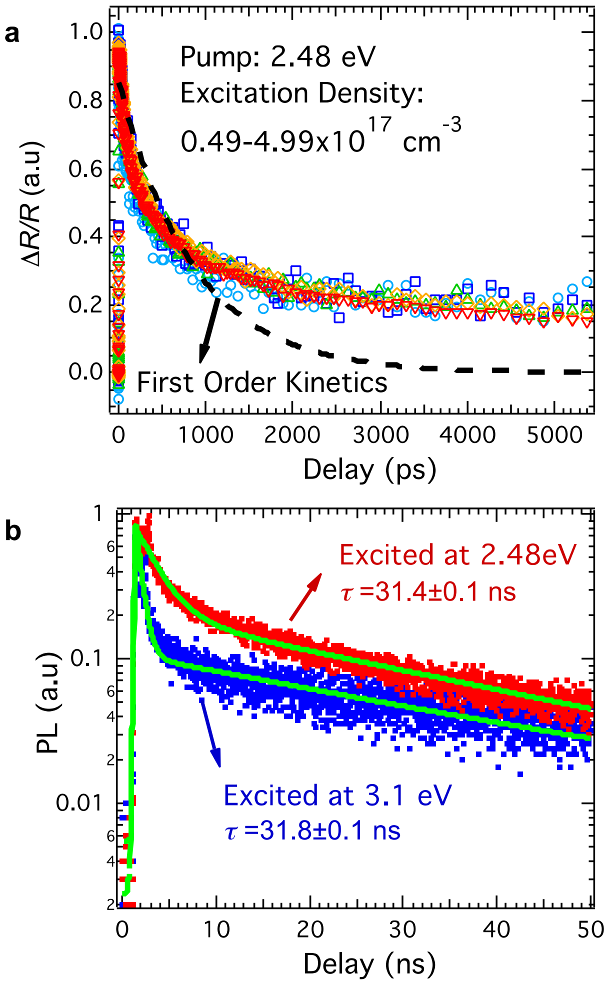

Supplementary Figure 3. TR kinetics and first order bulk recombination. (a) TR kinetics recorded at 2.38 eV for different intensities and the best fitting curve based on first order carrier recombination model. The large disagreement between TR kinetics and fitting curves suggests the incorrect fitting model. (b) The photoluminescence (PL) decay kinetics of the single crystal excited at 2.48 and 3.10 eV. The PL decay curves show two decay components. The fast component is attributed to the surface recombination that depends on the pump penetration depth (or pump energy). The slow component is due to the first order bulk carrier recombination that is independent of the pump penetration depth. The time constant of the slow decay component is determined from a single-exponential fit of the PL tail and indicated in the figure.

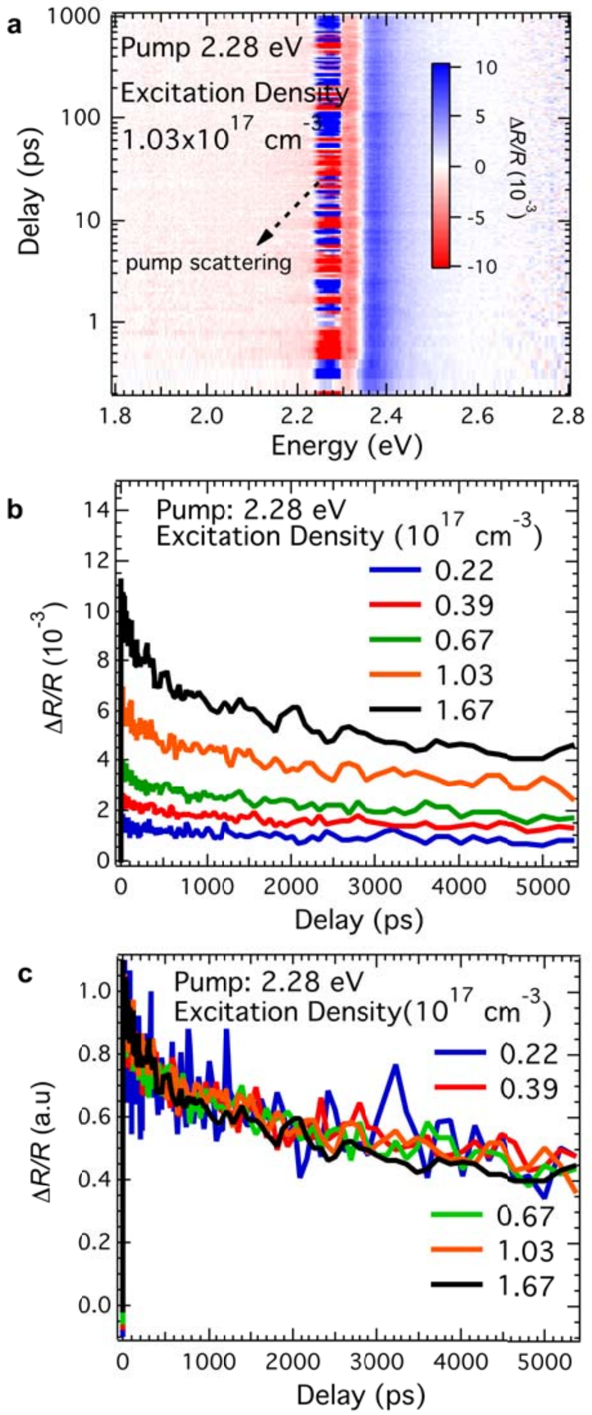

Supplementary Figure 4. TR measurement for the sample pumped at 2.28 eV. (a) Pseudocolor representation of the transient reflection spectra of the perovskite single crystal pumped at 2.28 eV. (b) The kinetics of the transient reflection recorded at 2.38 eV for different excitation densities. (c) The normalized kinetics shown in panel b.

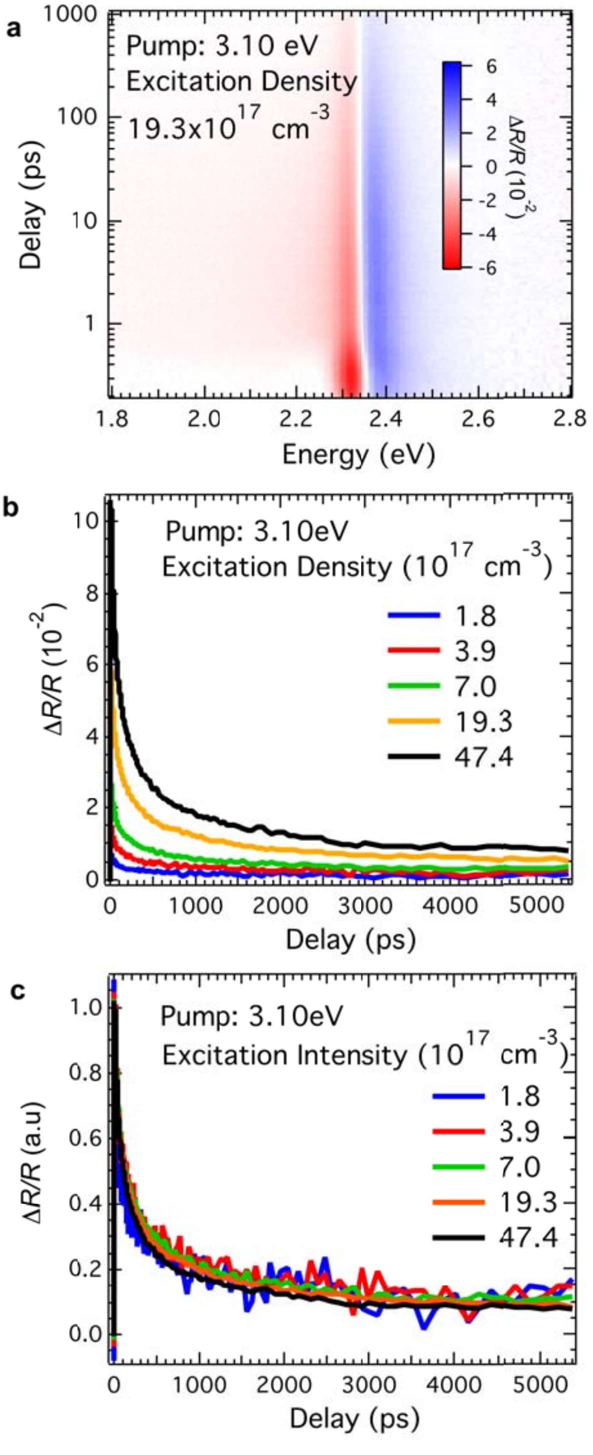

Supplementary Figure 5. TR measurement for the sample pumped at 3.10 eV. (a) Pseudocolor representation of the transient reflection spectra of the perovskite single crystal pumped at 3.10 eV. (b) The kinetics of the transient reflection recorded at 2.38 eV for different excitation density. (c) The normalized kinetics shown in panel b.

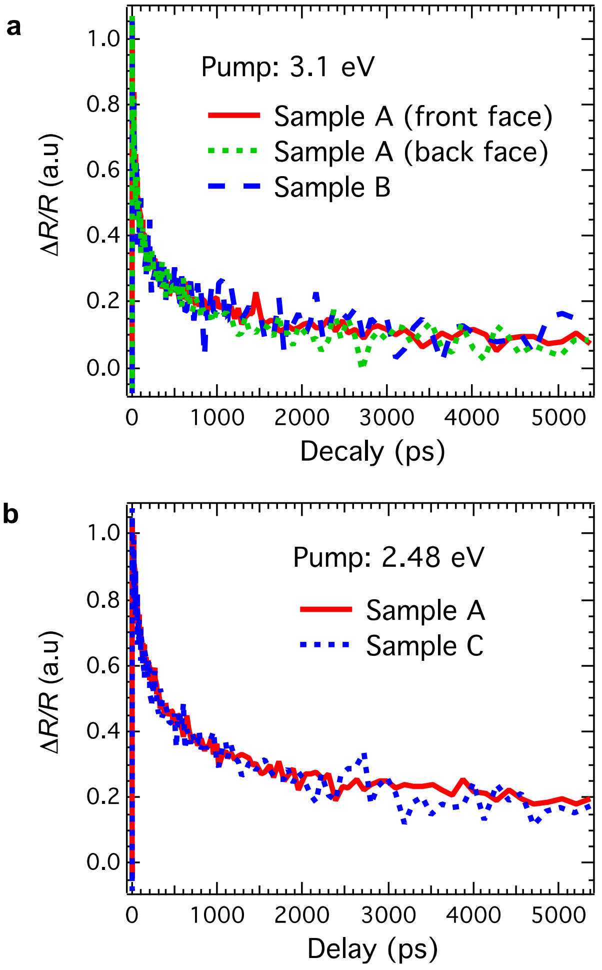

Supplementary Figure 6. Reproducibility test. (a) The normalized TR kinetics of two different samples pumped at 3.1 eV. Sample A is the one used for the experiment in main text. Sample B is a different single crystal. For sample A, the measurements were conducted on the front and back surfaces. The side surface is too small for optical measurement. (b) The normalized TA kinetics of two different samples pumped at 2.48 eV. Sample C is a third single crystal. This figure shows that the kinetics is only dependent on the pump energy. The consistence TR kinetics for different samples or different surfaces demonstrates the high reproducibility of our measurement and also confirms the high quality of our samples.

Supplementary Table.

Supplementary Table 1. List of Parameters for the absorption coefficient fitting shown in Fig. 1 in the main text.

E_g (eV)	R_{ex}(meV)	σ_c (meV)	σ_{ex}(meV)	$A(10^4 cm^{-1})$
2.394±0.0005	41.6±0.01	22.9±0.05	29.5±0.02	5.23±0.0008

In supplementary Table 1, E_g is bandgap, R_{ex} is exciton binding energy, σ_c(meV) and σ_{ex}(meV) are the standard deviation of the Gaussian broadening functions for continuum and excitonic absorption, respectively. A is the constant in Equation 1 in the main text, related to the transition matrix element.
